# Hyperferritinemia without iron overload in patients with bilateral cataracts: a case series

**DOI:** 10.1186/1752-1947-5-471

**Published:** 2011-09-21

**Authors:** Arne Kröger, Esther B Bachli, Andrew Mumford, Christoph Gubler

**Affiliations:** 1 Clinic and Polyclinic of Internal Medicine, University Hospital of Zurich, Rämistrasse 100, CH-8091 Zurich, Switzerland; 2 Department of Medicine, Uster Hospital, Brunnenstrasse 42, CH-8610 Uster, Switzerland; 3 Bristol Heart Institute, University of Bristol, Bristol, BS2 8HW, UK; 4 Clinic of Gastroenterology and Hepatology, University Hospital of Zurich, Rämistrasse 100, CH-8091 Switzerland

## Abstract

**Introduction:**

Hepatologists and internists often encounter patients with unexplained high serum ferritin concentration. After exclusion of hereditary hemochromatosis and hemosiderosis, rare disorders like hereditary hyperferritinemia cataract syndrome should be considered in the differential diagnosis. This autosomal dominant syndrome, that typically presents with juvenile bilateral cataracts, was first described in 1995 and has an increasing number of recognized molecular defects within a regulatory region of the L-ferritin gene (*FTL*).

**Case presentation:**

Two patients (32 and 49-year-old Caucasian men) from our ambulatory clinic were suspected as having this syndrome and a genetic analysis was performed. In both patients, sequencing of the *FTL *5' region showed previously described mutations within the iron responsive element (*FTL *c.33 C > A and *FTL *c.32G > C).

**Conclusion:**

Hereditary hyperferritinemia cataract syndrome should be considered in all patients with unexplained hyperferritinemia without signs of iron overload, particularly those with juvenile bilateral cataracts. Liver biopsy and phlebotomy should be avoided in this disorder.

## Introduction

Hereditary hyperferritinemia cataract syndrome (HHCS) is a rare autosomal dominant genetic disease, which was first described in 1995 independently by the groups of Bonneau [1] and of Girelli [2]. They reported two families in whom elevated serum L-ferritin concentration without iron overload, presenting with juvenile bilateral cataracts, was inherited as an autosomal dominant trait [1,2]. Cataracts comprise crystalline deposits of L-ferritin. The underlying molecular defect in both the early reports of HHCS was identified as point mutations in the 5' untranslated region (5'UTR) of the L-ferritin gene (*FTL*), in the region corresponding to the iron-responsive element (IRE) of L-ferritin messenger ribonucleic acid (mRNA) [3,4]. These mutations lead to loss of suppression of L-ferritin mRNA translation by the iron-dependent iron regulatory protein (IRP) leading to dysregulated expression of the L-ferritin protein. Since these early reports, a series of other point mutations and short deletions of L-ferritin IRE associated with HHCS have been reported.

In 2000, Rososchova *et al. *measured serum ferritin concentrations in 135 Swiss patients with bilateral operated cataracts before the age of 51 to detect HHCS. However, no patients with HHCS were identified. This led those authors to postulate that HHCS is so rare that it might not exist in Switzerland [5]. We describe, to the best of our knowledge, the first two cases of HHCS in Switzerland, both with proven mutations in *FTL*. We also review key aspects of the metabolism of cellular iron and ferritin synthesis and we discuss the pathophysiology of HHCS.

## Case presentations

### Patient 1

A 32-year-old Caucasian man from Switzerland was referred for further evaluation of an elevated serum ferritin, the test for which was ordered because of tiredness. His serum ferritin concentration at presentation was markedly elevated at 1314 μg/L (normal range 30-400 μg/L), but the serum transferrin saturation of 23.3% was within our laboratory reference interval (normal range 15-50%).

Our patient had no history of alcohol abuse or other metabolic diseases and no family history of hereditary hemochromatosis (HH). Clinical examination revealed no abnormalities. Further laboratory evaluation showed normal liver enzymes and normal hematological parameters.

Genetic tests for HH showed a heterozygous H63D substitution in the *HFE *gene but wild-type sequence at the *HFE *C282Y locus. The histology of a liver biopsy specimen was normal, and did not show iron accumulation or steatosis. Together, these findings exclude a diagnosis of HH.

**Figure 1:**
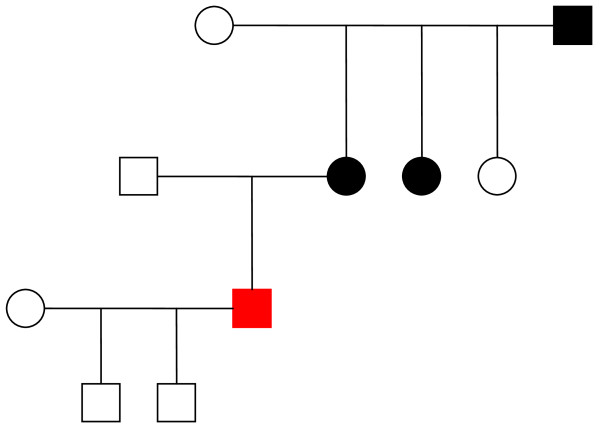
**Pedigree of the Patient 1**. Pedigree of patient 1. (circles - females, squares - males, black - affected members, red - patient 1)

**Figure 2:**
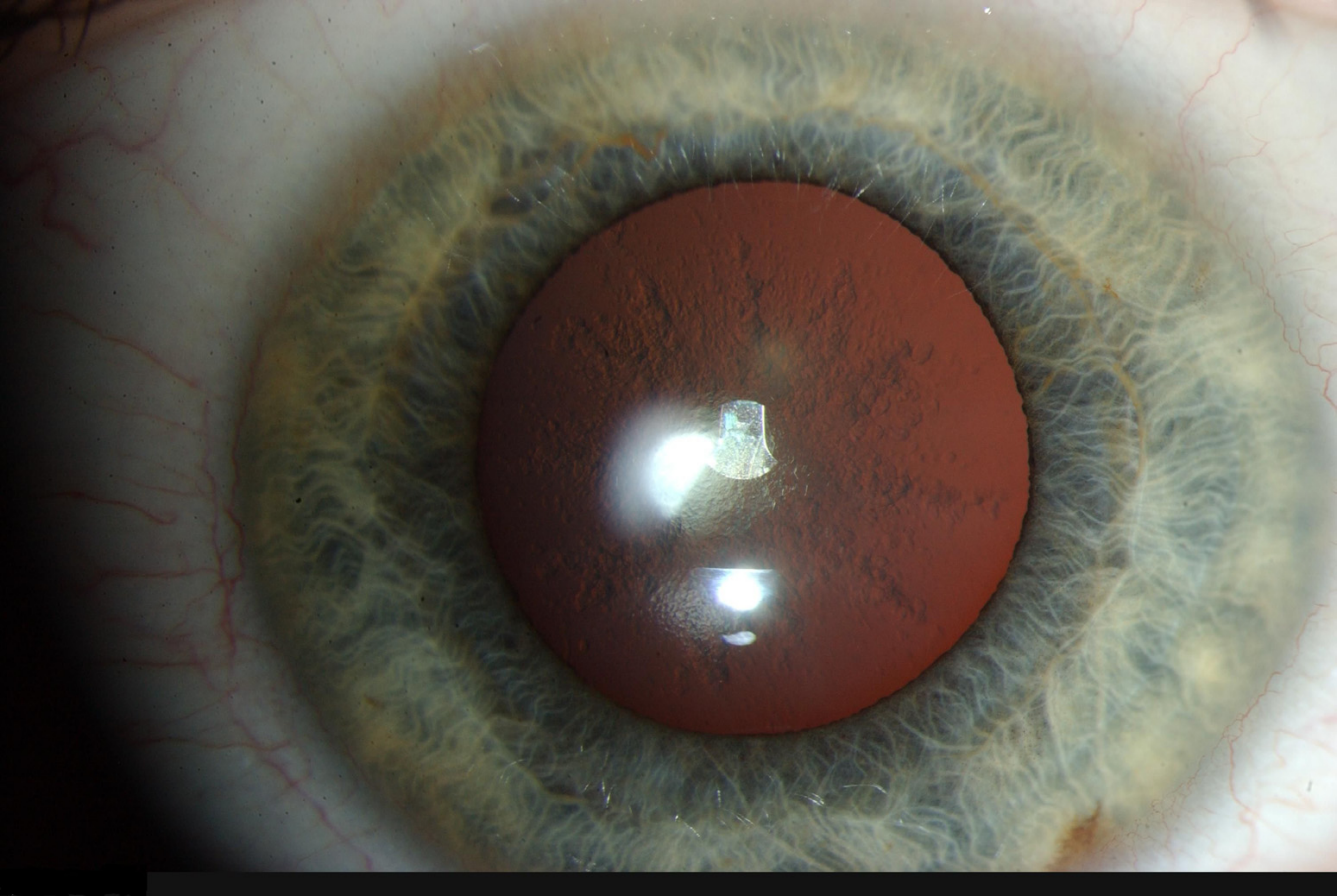
**Slit-lamp examination of the Patient 1**.

Upon further evaluation, our patient revealed a history of bilateral cataracts at four years of age. His mother, his maternal aunt and maternal grandfather had all had nuclear cataracts at an early age (Figure 1). Slit-lamp examination, direct illumination and retro-illumination of his lenses showed scattered, radially oriented flecks and crystalline deposits in both lenses (Figure 2). Sequencing of the *FTL *gene using a previously described method [6] showed a heterozygous c.33 C > A transversion in the IRE within the *FTL *5'UTR. This mutation has been previously associated with the HHCS phenotype [6].

### Patient 2

A 49-year-old Caucasian man was referred to our department for further examination of a markedly elevated serum ferritin concentration, of 2012 μg/L (normal range 30-400 μg/L). This had been identified as an incidental finding during the investigation of an allergy. His serum transferrin saturation of 29% was within the normal range. His liver enzymes and hematological evaluation were normal. The *HFE *gene showed wild-type sequence and an abdominal ultrasound showed normal liver echotexture. Our patient reported bilateral lens replacements 20 years ago due to bilateral juvenile cataracts. His family history was not available because he was an orphan. Deoxyribonucleic acid (DNA) sequencing of the *FTL *5'UTR revealed a heterozygous c.32G > C transversion. This mutation is also known to be associated with the clinical phenotype of HHCS [7].

## Discussion

Hereditary hemochromatosis (HH) is the most frequent treatable cause of hereditary iron overload in Caucasian patients (homozygosity in three to five out of a thousand). Since the initial manifestations of HH are frequently non-specific (for example tiredness and arthritis), serum ferritin is a frequently requested investigation in otherwise healthy patients. In some countries, measuring serum ferritin concentration has been proposed as a method of large-scale screening for HH, as iron overload in this disorder can be effectively controlled with phlebotomy if diagnosed before the onset of liver cirrhosis.

In our patients the reasons for requesting serum ferritin tests could not readily be explained. A high ferritin value and a normal transferrin saturation in an otherwise healthy young adult virtually excludes iron overload. In recent years, other rare disorders with or without late onset iron overload have been described and must be considered. One such disorder is autosomal dominant type A ferroportin disease, which presents with a low or slightly elevated transferrin saturation and tissue iron overload. Additionally, a number of rare autosomal recessive disorders causing iron overload are recognized, including aceruloplasminemia and atransferrinemia, which was first described in 1961 [8]. Both disorders are characterized by microcytic anemia and variable transferrin saturations. Aceruloplasminemia or hypoceruloplasminemia have additional features such as diabetes and neurological symptoms, such as cerebellar ataxia, dementia or extrapyramidal symptoms. None of these disorders of iron metabolism are associated with congenital or juvenile nuclear cataracts, which is a unique feature of HHCS.

In healthy individuals, the serum ferritin concentration correlates well with body iron stores. Serum ferritin is a byproduct of intracellular ferritin synthesis [9]. Ferritin is arranged in a particular way in order to create a cavity capable of storing up to 4500 Fe3+ ions as an inorganic complex [10,11]. As an intracellular iron storage molecule, it is a heteropolymer composed of 24 H and L subunits, variously assembled. Serum ferritin, on the other hand, consists mainly of L subunits, which can also be glycosylated (G).

The three different subunits composing the proteinous shell of human ferritin, L, H and G, arrange to form different isoferritins. The intracellular ferritin contains mostly L and H subunits. Serum ferritin consists of L and G subunits [3,10]. Ferritin synthesis is regulated by the availability of iron. An interaction between the IRP and the IRE of the *FTL *gene controls the translation of the L-ferritin gene. The IRE is a non-coding stem loop sequence located on the 5'UTR of the L-ferritin mRNA.

In the presence of abundant cellular iron there is a structural change in the IRP, that prevents the IRP from binding to the IRE, and ferritin synthesis will proceed. When there is a shortage of cellular iron, there is no relevant structural change and IRP binds to IRE and ferritin translation is inhibited [1,11-14].

In 1995, Bonneau *et al. *speculated that the reason for the accumulation of L-ferritin in HHCS is a mutation on the IRE coding region of L-ferritin [1]. In 1995, two groups in Italy and France simultaneously described the first two point mutations in the IRE of L-ferritin gene [3,4]. These mutations all change the structure of the IRE in a way which reduces or abolishes binding to the IRP. This leads to unregulated translation of the L-ferritin gene and consequently elevated levels of circulating L-ferritin [1,3,12,14,15].

Direct DNA sequencing was initially used to identify mutations in *FTL *and most of the know mutations are still detected by direct DNA sequencing. Another, faster method is double-gradient denaturing gradient gel electrophoresis, which is able to detect the mutations in a single run [6,16].

A distinguishing feature of HHCS is bilateral juvenile cataracts, which have an unusual morphology. They are described as "sunflower-type" morphology or "breadcrumb-like" [14]. The opacities consist of abundant L-ferritin protein. The precise mechanism by which this occurs is unclear. Lens opacities might be caused by a yet unknown interaction between L-ferritin and the lens proteins, or by a disturbed metabolism of L-ferritin within the lens [17]. The high protein concentration in the lens, the slow turnover of mature lens fibers after formation and the surrounds of the avascular lens may also be involved in the interaction. No involvement of organs other than the eye has been reported in patients so far [18]. Ferritin levels in HHCS can exceed values over 6000 μg/L without any correlation to the severity of the affected lens.

The prevalence of HHCS in different populations is unknown. A number of reports, mostly case reports, have previously been published [2,3,9,11,12,14,15].

In 2000 Rosochova *et al. *postulated that there were no HHCS cases in Switzerland. Over four years, between 1995 and 1998, 3000 patients with cataract operations were screened for HHCS. 135 patients were younger than 51 years and 19 of these had nuclear cataracts. In 15, serum ferritin and transferrin saturation could be measured. In two cases with elevated serum ferritin level (267 μg/L and 416 μg/L) and a positive family history for cataracts, further genetic analysis for HHCS was performed. DNA sequencing of the 5'UTR of L-Ferritin mRNA showed a normal nucleotide sequence in the whole region in both patients [5].

## Conclusion

We describe two unrelated patients in Switzerland with confirmed HHCS. High ferritin values in the absence of liver disease or any other disease, together with nuclear bilateral juvenile cataracts with or without a family history for juvenile cataracts, prompted this diagnosis. It is important to inform the patient and his or her family about the disease in order to prevent further evaluation for iron overload. Genetic confirmation should be obtained except in typical cases. Typical cases with otherwise unexplained hyperferritinemia presenting with autosomal dominant juvenile cataracts can be adequately diagnosed with a medical history and biochemical analyses. Nuclear cataracts can be treated with lens replacement therapy.

## Consent

Written informed consent was obtained from the patients for publication of this case report and any accompanying images. A copy of the written consent is available for review by the Editor-in-Chief of this journal.

## Competing interests

The authors declare that they have no competing interests.

## Authors' contributions

AM performed the sequencing of the *FTL *gene in both patients. CG and EB interpreted patients' history and data and, together with AK, were the major contributors in writing the manuscript. All authors read and approved the final manuscript.
